# Ion Chromatography: From Anions to Metals

**DOI:** 10.6028/jres.093.097

**Published:** 1988-06-01

**Authors:** William F. Koch

**Affiliations:** Center for Analytical Chemistry, National Bureau of Standards, Gaithersburg, MD 20899

From its humble beginnings in 1975 [1], ion chromatography has grown and expanded its scope to the extent that the term ion chromatography may no longer adequately describe the technique. As initially conceived, ion chromatography was configured with an ion exchange separation, followed by an ion suppression system to permit electrolytic conductivity detection of the analyte without overwhelming background from the eluent. This patented configuration found its best application in the determination of anions at trace (1–100 µg/mL) levels, an area long in want of a fast, accurate and reliable method. Some cations, specifically the alkali and alkaline earth metals, were also determinable in the early days, but it was the capability to determine anions that drove the technique’s popularity.

Soon, variations to the original concept were introduced, notably suppressorless or single column ion chromatography [2], and new types of detectors for ion chromatography. Amperometric and uv/visible spectrometric detectors have gained wide acceptance, especially in the determination of metals. Considerable effort was put into resin research to improve the sensitivity and resolution of the technique and to extend its capabilities to more ionic species. In some cases, ion exchange was no longer the dominant retention mechanism for the separation of various species. Hence, the term ion chromatography now is used to categorize any self-contained procedure based on chromatographic separation of ions followed by detection and quantification.

To typify the advances made in ion chromatography by noting recent work in the field at NBS, the detection limits for anion ion chromatography have dipped below 1 ng/mL for species such as sulfide and cyanide with amperometric detection, without preconcentration [3,4].

In other work, a method has recently been developed to determine alkali and alkaline earths in the pg/mL range with in-line preconcentration [5]. During the development of this method at NBS, the factors investigated included: composition of the eluents, interference due to impurities in the delivery liquid and the possible means of eliminating them with a laboratory-packed trap column, and a comparison of suppression efficiencies between two types of suppressors. This method was used for the determination of Mg^+2^ and Ca^+2^ in SRM 2694, Simulated Rainwater, with precisions and accuracies better than 2% relative [5].

The frontier for ion chromatography is transition metal analysis with ongoing research along many fronts. New resins are being introduced and are being coupled to exotic detectors. The era of “hyphenated” techniques is burgeoning with ion chromatography showing up as the front-end separation method of choice. Recent work at NBS has coupled ion chromatography with direct current plasma emission spectrometry for the determination of phosphorus in copper-based alloys [6].

Future challenges for ion chromatography will be to enhance further its capabilities through improved speed, sensitivity, and resolution. Speciation of metals as a function of oxidation state and complexation will be an important goal. Innovative resins and a new generation of detectors will have to be developed. Fundamental research into the retention mechanisms leading to the separation will be essential.

In conclusion, ion chromatography has proven itself an invaluable tool to analytical chemistry. Its ultimate advantages will lie in its versatility and its capacity for ultratrace analyses with minimal contamination and total automation.
